# Exploring the relationship between childhood trauma and internet addiction among nursing students: a latent profile and mediation analysis

**DOI:** 10.3389/fpsyt.2026.1734868

**Published:** 2026-02-25

**Authors:** Ying Li, Jieling Huang, Liuliu Kong, Yijun Lyu

**Affiliations:** 1 College of Sports Science, Jishou University, Jishou, Hunan, China; 2 Faculty of Health Sciences and Sports Education, Macau PolytechnicUniversity, Macao, Macao SAR, China; 3 Operating Room, Nanjing Gaochun People’s Hospital, Nanjing, Jiangsu, China

**Keywords:** alexithymia, childhood trauma, internet addiction, mental health, nursing education

## Abstract

**Background:**

Improving Internet addiction among nursing students is of great significance to the future development of the nursing industry. Previous studies have proved that childhood trauma is closely related to Internet addiction. However, the direct relationship between alexithymia and childhood trauma and Internet addiction has not been fully explored. The aim of this study is to identify different subgroups of nursing students based on their childhood trauma and to examine the mediating role of alexithymia between childhood trauma and Internet addiction.

**Method:**

From April to May 2025, 3,697 nursing students were recruited as samples from Shandong, Hubei, Hunan, and Henan provinces in China by convenient sampling. This survey collected social demographic data. Including The Childhood Trauma Questionnaire - Short Form (CTQ-SF), the Toronto Alexithymia Scale (TAS-26), and the Internet addiction Scale. Potential profile analysis was used to determine the potential categories of childhood trauma characteristics of nursing students, and Pearson correlation analysis, Bayesian factor robustness analysis and mediation analysis were used to determine the potential relationships among variables.

**Result:**

LPA identified three distinct groups based on their dominant usage: low (77.4%), medium (19.5%), and high (3.1%). In the relationship between childhood trauma and Internet addiction based on potential profile analysis, alexithymia has a significant mediating effect (SE = 0.442,95%CI = 0.095, 1.824; SE = 0.219, 95%CI = 0.093, 0.962).

**Conclusion:**

There is heterogeneity in childhood trauma among nursing students. Alexithymia plays an important mediating role in the relationship between childhood trauma and Internet addiction. It is suggested that nursing educators pay attention to the differences in childhood trauma among nursing students, provide corresponding psychological counseling for different students, improve them, thereby alleviating Internet addiction among nursing students and promoting their mental health.

## 1 Introduction

While judicious internet use enhances communication efficiency and facilitates knowledge acquisition, thereby supporting human development, its multifunctional nature simultaneously engenders problematic usage patterns that prove challenging to regulate. Notably, excessive engagement with internet-based technologies may impede developmental progress, compromise mental health, and impair social functioning, with severe cases potentially culminating in behavioral addiction (1, 2). Internet addiction has emerged as a prevalent public health concern among adolescents and young adults in recent decades. Clinically defined as an inability to control internet use accompanied by maladaptive behavioral patterns, internet addiction may lead to clinically significant impairment or distress (3, 4). Substantial evidence links internet addiction to multiple adverse health outcomes, including: (a) deteriorated mental health status (particularly anxiety and depression), (b) reduced physical activity levels, (c) altered dietary patterns, (d) cognitive impairment, (e) poor academic performance, and (f) increased risks of obesity and diabetes (5–7). The internet addiction phenomenon among nursing students deserves attention as it may affect their academic and future professional performance. Internet addiction is characterized by an uncontrollable desire to go online, which can cause harmful consequences in all aspects of life (8). Previous studies have found that Internet addiction among nursing students is at a moderate level (8, 9). The occurrence of Internet addiction among nursing students can reduce academic performance, interfere with daily life activities, increase feelings of loneliness and isolation, and lead to personality distortion (10), which is not conducive to the development of the nursing profession. Understanding the factors influencing Internet addiction among nursing students is helpful for formulating targeted intervention measures, thereby promoting the development of talents in the nursing profession. Current research on the factors of Internet addiction among nursing students mainly focuses on external factors, such as sociodemographic characteristics and Internet usage time (11). This attention largely neglects the crucial role of internal personal traits. Therefore, this study aims to further expand the existing knowledge system of Internet addiction among nursing students by investigating the influence of the intrinsic personal traits of nursing students on their Internet addiction.

Childhood trauma plays an indispensable role in the formation and development of Internet addiction behaviors (12, 13). Childhood trauma is defined as physical or emotional abuse, neglect, and sexual abuse experienced by an individual before the age of 16 (14). Previous studies have shown that childhood trauma is closely related to Internet addiction. Individuals who have suffered childhood trauma are more likely to develop Internet addiction, and factors such as the type of trauma, the severity of trauma, and cultural background may play a moderating role in this (15, 16). Childhood trauma encompasses both physical and psychological aspects. The emotional and affective neglect and abuse that an individual experiences during childhood can have a greater impact on their bad behavior in adulthood than physical harm. At every stage of life, there is a significant correlation between traumatic experiences and mental illness.

Similarly, traumatic experiences can effectively predict an individual’s Internet addiction behavior (17). Not only child abuse, but also many other types of trauma can increase an individual’s risk of addictive behavior (18). The compensatory Internet usage model holds that individuals may compensate for unmet needs in real life in the online world (19), and childhood trauma can undermine the acquisition of individual emotional regulation ability (20). When negative emotions cannot be effectively vented in real life, individuals may use the Internet as an escape or coping strategy to mitigate the impact of negative emotions. If this continues for a long time, it may lead to Internet addiction (19). Latent Profile Analysis (LPA) is a human-centered statistical method that classifies individuals based on common attributes, behaviors, or characteristics of individuals and occupations, which stem from their responses to specific observations (21). The advantage of applying LPA in this study lies in its ability to provide a more precise understanding of childhood trauma among nursing students by identifying different subgroups that might be overlooked in the overall score. This method provides valuable insights for improving the pattern of Internet addiction among nursing students and informs for future research and the development of targeted intervention measures.

The main feature of alexithymia is the difficulty in recognizing, distinguishing and expressing emotions (22). It is a common psychosomatic phenomenon that brings a series of problems to the healthy growth of teenagers (23). The prevalence of alexithymia is 10% in the general population and 36% among adolescents (24, 25). Alexithymia is significantly positively correlated with Internet addiction (26, 27). Individuals with alexithymia have difficulty recognizing emotions, expressing them in appropriate words, and understanding the reasons for others’ emotional changes, which leads to a series of problems in their interpersonal relationships, such as social phobia and social avoidance (28). The indirectness of online social interaction to some extent compensates for the deficiency of alexithymia in emotional cognition and expression ability, but it also makes alexithymia overly dependent on the Internet, making the phenomenon of their detachment from the real society more serious (29).

Childhood trauma is considered to be the main risk factor of alexithymia, which hinders the ability to identify and express emotions, thus increasing the psychological distress of adolescents, and is closely associated with the occurrence and development of psychotic symptoms (30). Alexithymia can increase the susceptibility of adolescents to Internet addiction through deficiencies in cognitive processing and emotional regulation (31, 32), and has been proven to be a significant positive predictor of Internet addiction. However, it remains unclear whether alexithymia plays a mediating role between childhood trauma and Internet addiction among nursing students.

**Figure 1:**
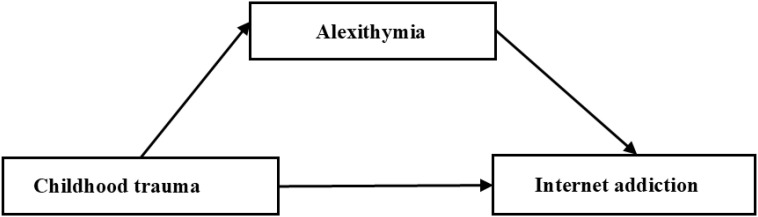
The conceptual model.

This study aims to explore the relationship between childhood trauma and Internet addiction among nursing students, emphasizing the mediating role of alexithymia. This study is based on the existing literature in several key fields (8, 11, 33). Although there is growing interest in childhood trauma (34, 35), research on nursing students remains limited. Furthermore, most of the current research both domestically and internationally tends to focus on the analysis at the group level, while neglecting the variability of individuals. This approach often fails to provide tailor-made guidance for personalized practices. To address this issue, we employed latent category analysis to determine the heterogeneity in childhood trauma among nursing students. This study employed a mediating model to analyze the mediating role of alexithymia between childhood trauma and Internet addiction. Based on these goals and theoretical frameworks, three hypotheses are proposed: H1: Childhood trauma is significantly associated with Internet addiction; H2: The heterogeneity of childhood trauma among nursing students can be identified through LPA. H3: Alexithymia plays a mediating role between childhood trauma and Internet addiction, **Figure 1**.

## 2 Materials and methods

### 2.1 Participants

In April and May 2025, this study recruited nursing students from 10 universities across Shandong, Hubei, Hunan, and Henan provinces using convenience sampling. Data were collected through anonymous self-administered questionnaires distributed by class teachers. Questionnaire distribution continued until no new data were generated for seven consecutive days, at which point data collection ceased. Of the 3,850 completed questionnaires, 153 were excluded due to missing items or uniform responses. Consequently, 3,697 valid responses were retained for analysis.

Inclusion criteria comprised: (1) Enrollment in a nursing degree program; (2) Mandarin language proficiency sufficient for unhindered communication; (3) Willingness to provide informed consent.

Exclusion criteria included: (1) A documented diagnosis of mental disorders; (2) Medical leave or academic suspension during the study period.

### 2.2 Measures

#### 2.2.1 Social demographic information

Social demographic information includes gender (male or female), age, living status (urban or rural), educational level and monthly household income.

##### 2.2.1.1 The childhood trauma questionnaire–short form

CTQ-SF (14), developed by Bernstein et al., is a 28-item self-report instrument designed to assess five dimensions of childhood trauma. The questionnaire consists of 25 clinical items evaluating trauma experiences and 3 validity items to detect potential response bias (e.g., minimization or denial of traumatic experiences). The CTQ-SF measures the following subscales, each comprising 5 items: Physical abuse, Emotional abuse, Sexual abuse, Emotional neglect, Physical neglect All items are rated on a 5-point Likert scale (1 = *never* to 5 = *always*). Notably, items 2, 5, 7, 13, 19, 26, and 28 require reverse-coding prior to analysis. Subscale scores range from 5 to 25, while the total score ranges from 25 to 125, with higher scores indicating more severe childhood trauma exposure. In this study, the CTQ-SF demonstrated excellent internal consistency, with a Cronbach’s α of 0.879 for the total scale.

##### 2.2.1.2 Toronto alexithymia scale

Toylor et al. compiled the TAS-26 in 1984 (36). Subsequently, in 1991, Chinese scholar Yao Fangchuan carried out the Chinese translation (37). This study adopted the Toronto Alexithymia Scale revised by Chinese scholar Professor Yao Shuqiao et al (38). The scale contains 26 questions and four dimensions: the ability to describe emotions, recognize and distinguish between emotions and body feelings, fantasy, and extroverted thinking. A 5-point scale is used, from 1 (strongly disagree) to 5 (strongly agree), with a total score ranging from 20 to 100. The higher the score, the more severe the alexithymia. In this study, the Cronbach’s α for the sample was 0.934.

### 2.3 Internet addiction

The Internet Addiction Scale was compiled by K.S. Yeong of the University of Pittsburgh (39). It has been widely used to measure Internet addiction (40). The scale consists of 20 items, each with five choices (almost none - always), assigned a score of 1-5. It includes four dimensions: network damage, compulsive symptoms, network relationship addiction, and the influence of destructive emotions. The subjects’ Internet use was determined according to the scale’s total score. The higher the score, the more serious the degree of addiction to the internet. The score of 20–49 was everyday Internet use, and the score of 50–100 was internet addiction. In this study, the Cronbach’s α for the sample was 0.961.

### 2.4 Statistical analysis

Descriptive statistics were first employed to analyze participants’ demographic characteristics, followed by Harman’s single-factor test to assess potential common method variance (CMV) (41). Pearson correlation analysis was then conducted to examine intervariable associations (42). Latent profile analysis (LPA) was performed to identify subgroups reflecting distinct patterns of childhood trauma. Model fit was evaluated using multiple indices, including entropy, Akaike information criterion (AIC), Bayesian information criterion (BIC), and sample-size-adjusted BIC (aBIC) (43). Univariate and multivariate analyses were further applied to determine factors associated with the derived LPA profiles. Additionally, Bayesian independent-samples t-tests were conducted to compare childhood trauma severity across LPA subgroups. To examine the mediating role of alexithymia in the association between childhood trauma and internet addiction, Hayes’ PROCESS macro (Model 4) in SPSS 26.0 was utilized. All statistical analyses were performed using SPSS 27.0 (IBM Corp., Armonk, NY, USA), Mplus (Version 7.4), and JASP (Version 0.19.3.0).

## 3 Results

### 3.1 Common method variance test

To assess potential common method bias, we performed Harman’s single-factor test by conducting exploratory factor analysis (EFA) with principal component extraction on all items measuring nursing students’ internet addiction, childhood trauma, and alexithymia (44). The results revealed 10 factors with eigenvalues >1, with the first factor accounting for 15% of the total variance - below the critical threshold of 40%. These findings suggest no substantial common method bias in the current study.

### 3.2 Sample characteristics

3,697 college students, among whom 82.6% were female (n = 3,052). **Table 1** lists other sociodemographic details. Significant differences were found in variables such as Education level and Family economic status in childhood trauma (see **Table 1**).

**Table 1:** Childhood trauma scores among different sociodemographic groups.

Characteristics		N (%)	Mean (± SD)	t/F	P
Gender	Female	3052(82.6%)	39.70 ± 10.692	-1.254	0.210
	Male	645(17.4%)	40.29 ± 12.333		
Age (Year)	≤18	1478(40%)	40.01 ± 10.829	2.595	0.075
	19-22	2108(57.0%)	39.77 ± 11.070		
	≥23	111(3.0%)	37.55 ± 11.629		
Education level	Vocational secondary school	1115(30.2%)	39.13 ± 10.848	14.058	**<0.001**
	College diploma	1102(29.8%)	38.91 ± 10.978		
	Undergraduate degree	1480(40%)	40.96 ± 11.025		
Residence	Rural area	1936(52.4%)	40.13 ± 11.074	1.941	0.052
	Urban area	1758(47.6%)	39.43 ± 10.898		
Family Economic Status	Very poor	605(16.4%)	39.89 ± 11.622	4.111	**0.006**
	poor	1610(43.5%)	40.40 ± 10.761		
	Moderate	898(24.3%)	39.49 ± 10.453		
	Good	576(15.6%)	38.61 ± 11.649		

Bold values indicate P< 0.05.

### 3.3 Pearson’s analysis of the correlation between childhood trauma, alexithymia, internet addiction

Childhood trauma was positively correlated with Alexithymia (r=0.044, P < 0.05), and Childhood trauma was positively correlated with Internet addiction (r=0.203, P < 0.05). Detailed results were provided in **Table 2**.

**Table 2:** Pearson’s analysis of the correlation between childhood trauma, alexithymia, internet addiction.

Variables	Childhood trauma	Alexithymia	Internet addiction	Gender	Age	Education level	Residence
Childhood trauma	–						
Alexithymia	0.044*	–					
Internet addiction	0.203**	0.374**	–				
Gender	0.090**	-0.034*	0.021	–			
Age	-0.147**	-0.031*	-0.025	0.103**	–		
Education level	-0.130**	0.046**	0.073**	0.089**	0.637**	–	
Residence	-0.018	-0.023	-0.032	0.030	-0.083**	-0.030	–
Family Economic Status	-0.131**	-0.034*	-0.046**	0.056**	0.057**	0.141**	0.293**

* P<0.05, **P<0.01, ***P<0.001.

### 3.4 Latent profile analysis of childhood trauma

**Figure 2:**
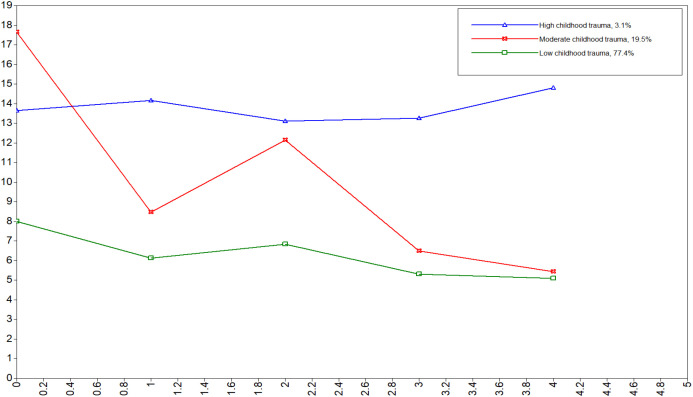
Estimated sample item means for the three latent profiles of childhood trauma.

To find the most suitable model for the data, the fitting indices of the first to fourth types of solutions were compared (see **Table 3**). Model 4 does not meet the fitting criteria when LMRTP is greater than 0.05. AIC, BIC, and aBIC decrease with the increase of categories. The better the model fits, although Model 3 has the lowest entropy among the other two models, it is still greater than 0.90, and the classification accuracy is good. Moreover, the classification of Model 3 can present the characteristics of childhood trauma of different types of nursing students more clearly and precisely. Therefore, Model 3 is determined as the best potential profile model. The groups were respectively the low childhood trauma group (3.1%), the moderate childhood trauma group (19.5%), and the high childhood trauma group (77.4%). **Figure 2** showed the estimated sample item means for the selected three-profile solution. Logistic regression analyses showed that gender, education level, family economic status were indicators of childhood trauma types (see **Table 4**).

**Table 3:** Fit statistics for latent profile models of childhood trauma.

Indices	Unconditional model			
	1-profile	2-profile	3-profile	4-profile
Fit statistics				
LL	-45333.074	-41114.328	**-39518.222**	-38234.794
AIC	90686.148	82260.656	**79080.444**	76525.587
BIC	90748.301	82360.100	**79217.180**	76699.615
aBIC	90716.526	82309.260	**79147.275**	76610.645
Entropy	–	0.999	**0.935**	0.946
BLRT	–	<0.001	**<0.001**	<0.001
LMRT	–	0.0034	**<0.0001**	0.395
Group sizes (%)				
C1	100%	3.7%	3.1%	2.5%
C2		96.3%	19.5%	2.7%
C3			77.873%	76.4%
C4				2.5%

LL, Log-likelihood; AIC, Akaike Information Criterion; BIC, Bayesian Information Criterion; aBIC, adjusted BIC; LMR, Lo–Mendell–Rubin likelihood ratio test.

Bold values indicate P< 0.05.

**Table 4:** Univariate and multivariate logistic regression results for predicting external features on the 3-class pattern.

Characteristics	Univariate analysis				Multivariate analysis			
	High childhood trauma VS Low childhood trauma		Moderate childhood trauma VS Low childhood trauma		High childhood trauma VS Low childhood trauma		Moderate childhood trauma VS Low childhood trauma	
	OR (95%CI)	*P*	OR (95%CI)	*P*	OR (95%CI)	*P*	OR (95%CI)	*P*
Gender (male as reference)								
Female	0.956(0.767-1.192)	0.688	0.184(0.126-0.270)	**<0.001**			0.180(0.122-0.265)	**<0.001**
Age (≥23 as reference)								
<19	2.540(1.406-0.589)	**0.002**	0.944(0.284-3.141)	0.926	1.673(0.882-3.174)	0.115		
19-22	1.362(0.754-2.462)	0.306	1.444(0.447-4.658)	0.539				
Education level (Undergraduate degree as reference)								
Vocational secondary school	2.405(1.971-2.934)	**<0.001**	0.443(0.252-0.778)	**0.005**	1.822(1.360-2.440)	**<0.001**	**1.286(1.029-1.606)**	**0.027**
College diploma	1.378(1.111-1.710)	**0.004**	1.035(0.685-1.563)	0.871	**0.402(0.196-0.824)**	**0.013**		
Residence (Urban area as reference)								
Rural area	0.928(0.787-1.094)	0.371	1.156(0.790-1.691)	0.456				
Family Economic Status (Good as reference)								
Very poor	2.365(1.760-3.178)	**<0.001**	1.773(0.979-3.209)	0.059	**2.284(1.672-3.120)**	**<0.001**		
Poor	1.427 (1.095-1.861)	**0.009**	0.937(0.544-1.613)	0.814	**1.328(1.006-1.751)**	**0.045**		
Moderate	1.133(0.844-1.522)	0.405	0.611(0.318-1.177)	0.141				

Bold values indicate P< 0.05.

**Figure 3:**
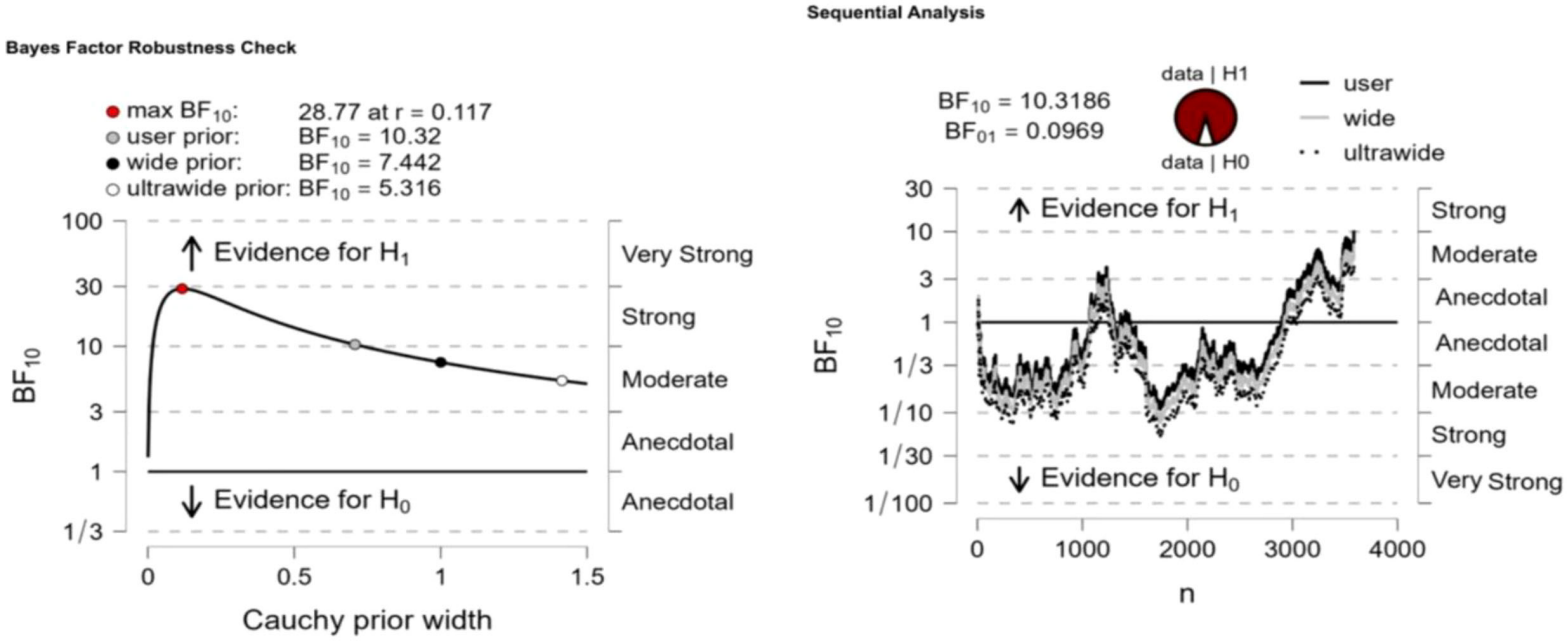
High childhood trauma VS low childhood trauma.

**Figure 4:**
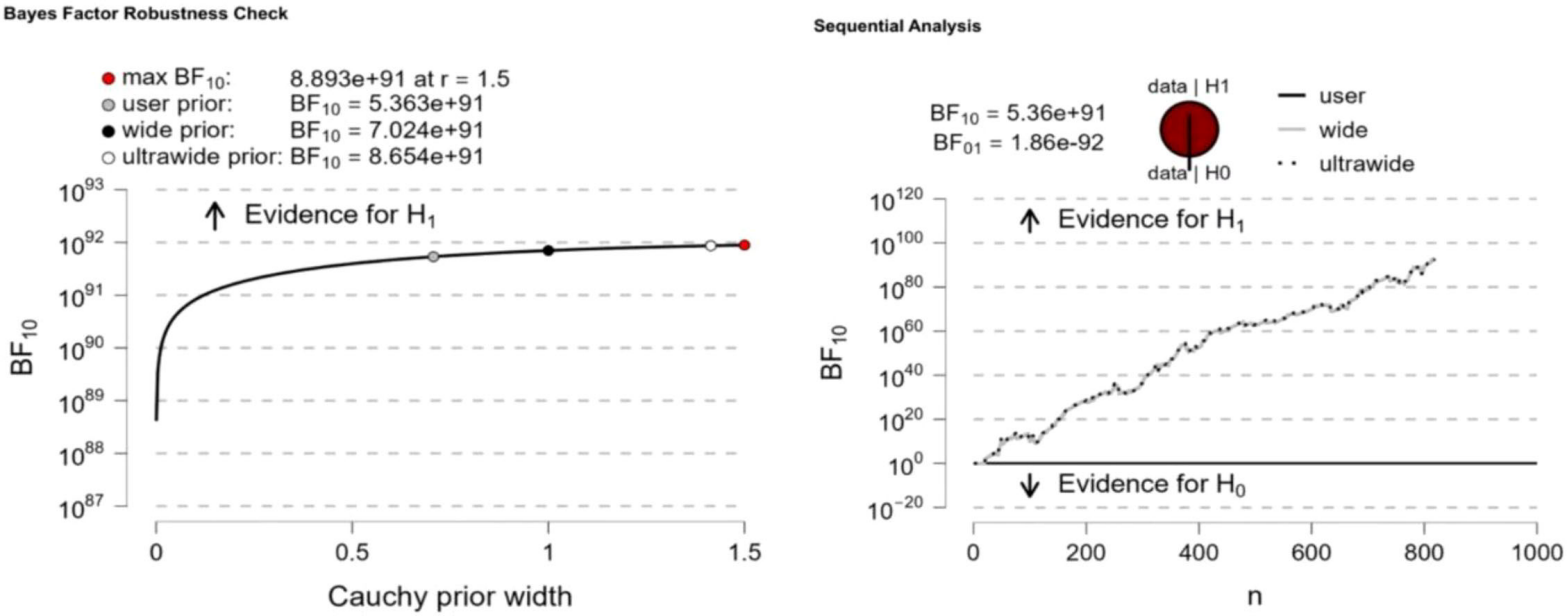
High childhood trauma VS moderate childhood trauma.

**Figure 5:**
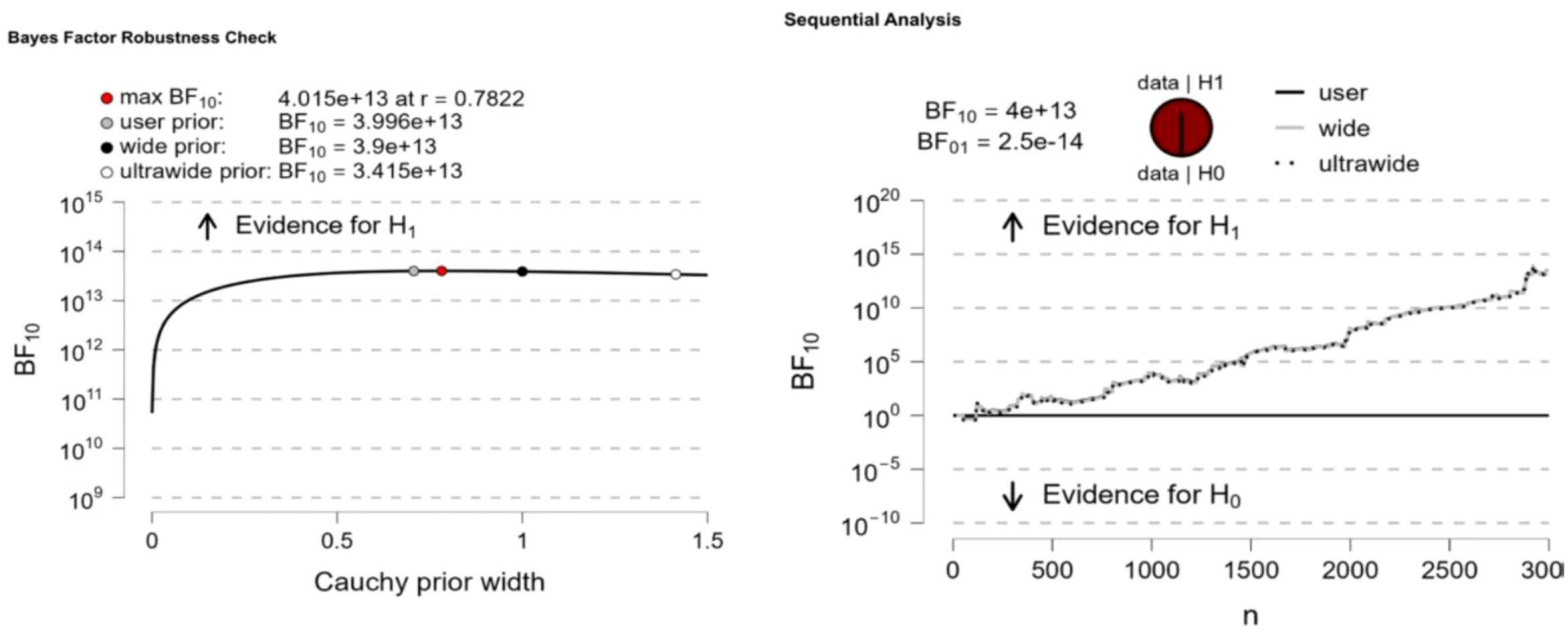
Moderate childhood trauma VS Low childhood trauma.

Significant differences in Internet addiction were observed between “high childhood trauma” and “low childhood trauma” (BF10 = 28.77), “high childhood trauma” and “moderate childhood trauma” (BF10 = 8.839e+91), and “moderate childhood trauma” and “low childhood trauma” (BF10 = 4.015e + 13). Bayesian factor robustness analysis confirmed these differences, and further details are shown in **Figures 3**–**5**.

**Figure 6:**
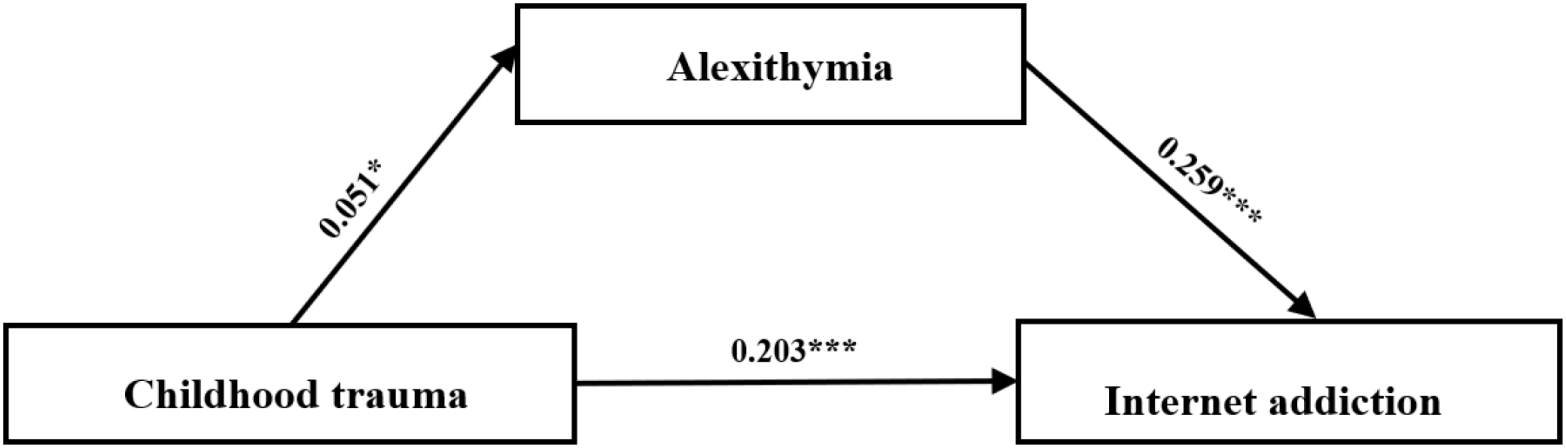
A mediator model with childhood trauma as the independent variable (X), alexithymia as the mediator variable (M), and internet addiction as the dependent variable (Y). Control variables are not shown in the figure for brevity.

After adjusting for general variables and designating moderate childhood trauma as the reference group, the analysis revealed the following 95% bootleg confidence intervals (**Tables 5**, **6**, **Figure 6**): indirect (0.095,1.824), direct (3.245,7.315), and total (4.029, 8.405). Research has found that alexithymia plays a significant mediating role and shows a sufficient mediating effect between moderate childhood trauma and low childhood trauma.

**Table 5:** The mediating role of alexithymia in nursing students between childhood trauma and internet addiction.

	Variables	Effect	SE	t	LLCI	ULCI
Total effects	Moderate childhood trauma	6.217	1.116	5.570	4.029	8.405
	Low childhood trauma	-1.663	0.462	-3.599	-2.569	-0.757
Direct effects	Moderate childhood trauma	5.280	1.038	5.086	3.245	7.315
	Low childhood trauma	-2.186	0.430	-5.085	-3.030	-0.199
Indirect effects	Moderate childhood trauma	0.937	0.442	–	0.095	1.824
	Low childhood trauma	0.523	0.219	–	0.093	0.962

**Table 6:** Mediation model test.

Variables	Internet addiction			Alexithymia			Internet addiction		
	β	SE	t	β	SE	t	β	SE	t
Childhood trauma	0.203	0.016	12.522***	0.051	0.024	2.122*	0.190	0.015	12.664***
Alexithymia							0.259	0.010	25.082***
Gender	0.063	0.470	0.134	-1.450	0.694	-2.089*	0.438	0.434	1.008
Age	-2.085	0.426	-4.899***	-2.972	0.639	-4.726***	-1.316	0.395	-3.337***
Education level	2.209	0.277	7.984***	2.283	0.409	5.582***	1.619	0.257	6.304***
Family Economic Status	-0.356	0.200	-1.779	-0.519	0.296	-1.751	-0.222	0.185	-1.201***
Residence	-0.486	0.370	-1.314	0.547	0.547	-1.026	-0.341	0.342	-0997
R^2^	0.059				0.013		0.196		
F	38.312***				248.573***		128.319***		

***P<0.001, *P<0.05

## 4 Discussion

This study took 3,697 nursing students from 10 universities as samples to investigate the interaction between childhood trauma, alexithymia and Internet addiction, as well as the variability of childhood trauma. All the hypotheses have been confirmed. Firstly, the research results show that there is a positive correlation between childhood trauma and Internet addiction (13), which is consistent with previous research findings. Adolescents who have experienced trauma in childhood may develop insecure attachments, which can lead to negative emotional and behavioral consequences (45). Teenagers who grow up in an unsafe family environment and experience childhood trauma may relieve negative emotions by overusing the Internet, eventually leading to Internet addiction. From the perspective of neurophysiology, family stressors (such as childhood trauma) can disrupt the stress response system of adolescents, alter neural structure and function, and increase susceptibility to Internet addiction (45, 46). This emphasizes the importance of improving childhood trauma and suggests that nursing educators actively identify childhood trauma among nursing students, prevent the occurrence of Internet addiction problems, and promote the healthy development of nursing students.

Consistent with the second hypothesis, LPA classified the samples into three distinct profiles: low childhood trauma, moderate childhood trauma, and high childhood trauma. More than half of the nursing students showed low childhood trauma, which is consistent with the results of earlier studies (47). Comparative analysis revealed that female nursing students were significantly more likely to report low childhood trauma. This may be attributed to greater societal acceptance and nurturing towards girls in Chinese culture. Additionally, girls’ generally compliant temperament and lower propensity for disruptive behaviors likely reduced caregiver antagonism, thereby decreasing their exposure to traumatic experiences. The comparative group analysis shows that low-income families are more prone to childhood trauma. Our research results are the same as those of previous studies (48). Children from low-income families are more likely to suffer emotional and physical neglect from their parents (49). Economic pressure leads to parents’ lack of energy. Low-income parents mostly engage in high-intensity and low-pay work and have no time to take care of their children when they return home. Due to deviations in educational concepts, low-income parents often only meet their children’s material needs and neglect their emotional needs (50).

The third hypothesis was supported by the significant mediating effect of alexithymia, indicating its crucial role in the association between childhood trauma and internet addiction. This finding aligns with the cumulative situational risk model, which posits that increased exposure to adverse childhood experiences elevates the likelihood of maladaptive outcomes in adulthood (51). Self-determination theory (52) provides a mechanistic explanation: Childhood trauma may compromise the fulfillment of three fundamental psychological needs—autonomy, relatedness, and competence—thereby increasing vulnerability to problematic internet use as a compensatory behavior.

Research indicates that individuals who have experienced childhood trauma often face diminished social support and limited parental social interaction, resulting in a lack of experience in expressing emotional experiences (53). Furthermore, family environments may not encourage the expression of emotions, leading to difficulties in describing, identifying, and differentiating emotions (54). Due to challenges in recognizing their own feelings and deficiencies in emotional expression skills, these individuals tend to focus more on physical discomfort rather than addressing their inner emotional needs when describing their discomfort (55). This obstacle in emotional expression hinders their ability to communicate effectively with others, ultimately impacting the development of interpersonal relationships. In this context, the virtual nature and convenience of the internet provide a platform for these individuals, who repress their emotions in real life, to release their feelings. This allows them to fulfill their emotional needs in a manner that minimizes direct conflict (55). However, this reliance on online platforms simultaneously heightens the risk of developing internet addiction. Furthermore, individuals who have suffered verbal abuse during childhood will have language-related brain regions affected, such as Broca’s and Wernicke’s areas, which in turn leads to a decline in language function and comprehension ability, and their emotional expression ability will also be affected to a certain extent (56). Secondly, psychological trauma experienced by children in their early years can affect an individual’s ability to regulate emotions, impair their imagination, cause a lack of lifelong pleasure, and lead to an insecure attachment style, thereby resulting in alexithymia (57, 58). Individuals with alexithymia have lower self-esteem levels and emotional control abilities than the general population. They have deficiencies in emotional expression, perception of emotions, and emotional imagination, which makes them more prone to negative emotions when frustrated in real social environments. At the same time, they have difficulties in social interaction and find it hard to establish a good social support system (27), thus seeking compensation through the Internet. It increases the risk of Internet addiction (59). Our research results further confirm the previous studies on the mediating role of alexithymia in childhood trauma and Internet addiction (33). Therefore, it is necessary to screen for childhood trauma among nursing students, carry out early intervention, and reduce the occurrence of Internet addiction among nursing students.

This study Outlines strategies for promoting caregiver growth in the field of mental health and emphasizes the importance of understanding the impact of different degrees of childhood trauma on Internet addiction. Taking “high childhood trauma” as a reference, we respectively explored the influences of low childhood trauma and moderate childhood trauma on Internet addiction among Internet care students. Nursing students who are regarded as having “ Moderate childhood trauma” have experienced childhood trauma, which causes them to bear excessive psychological pressure and makes them more prone to sub-health problems. This also suggests that nursing educators should pay attention to those with early traumatic experiences. When necessary, “psychodrama” (60) or the intervention of a psychologist should be adopted to reduce the harm caused by childhood trauma and thereby improve the occurrence of Internet addiction. For nursing students classified as having “low childhood trauma”, their probability of developing Internet addiction is lower than that of those with high childhood trauma. However, alexithymia still plays a certain role. People with alexithymia have obvious deficiencies in emotional processing ability. When it comes to interpersonal communication, there are difficulties in adaptation and they are unable to establish the friendships they need (61). The use of the Internet precisely meets the extensive interpersonal communication needs that cannot be fulfilled in real life, becoming the realistic basis for college students’ tendency towards Internet addiction. For nursing students with alexithymia, nursing educators provide emotional support and psychological counseling to reduce the occurrence of alexithymia. On the other hand, the mindfulness level of nursing students can lower negative cognition and improve their Internet addiction (62).

## 5 Limitations

This study has certain limitations. Although we conducted a large-sample study, the convenient sampling method may not fully represent nursing students from different regions and organizations, which affects the generalization of the current results to all nursing professions. In future research, larger samples can be taken, and the profiles of different regions, cultures and countries can also be investigated to explore possible differences and commonalities. Secondly, since our data was collected through self-reports and there is a social desirability bias, it is difficult to generalize the results. Finally, we recognized the limitations of cross-sectional design in determining causal relationships and plan to use longitudinal design in future studies to better understand the temporal sequence and causal relationships among variables.

## 6 Conclusion

This study ultimately established a positive correlation between childhood trauma and Internet addiction, with alexithymia serving as a mediating variable in this dynamic. Furthermore, nursing students have revealed the direct relationship between different groups and Internet addiction based on the heterogeneity of LPA usage, and have put forward different suggestions for different groups. The findings of this study provide crucial guidance for nursing educators and administrators. By categorizing nursing students into distinct risk groups based on their childhood trauma experiences, educators and managers can promptly implement targeted interventions. In particular, for students in the moderate childhood trauma group, educators should maintain heightened vigilance, closely monitor their physical and mental well-being, and strengthen psychological support. For those in the high childhood trauma group, nursing educators and administrators should actively adopt personalized and tailored measures, such as psychotherapy, to mitigate the negative impacts of childhood trauma. This approach will enhance the overall well-being of nursing students and ultimately contribute to the broader healthcare system.

## Data Availability

The original contributions presented in the study are included in the article/supplementary material. Further inquiries can be directed to the corresponding author.
